# Survival and predictors of neonatal mortality: a hospital-based retrospective follow-up study from Addis Ababa, Ethiopia

**DOI:** 10.7717/peerj.21473

**Published:** 2026-07-22

**Authors:** Ruth Woldeyohannes Yirgu, Zekarias Amdemariam, Nebiyou Fasil, Firehiwot Workneh

**Affiliations:** 1 Zewditu Memorial Hospital, Addis Ababa, Ethiopia; 2 Addis Continental Institute of Public Health, Addis Ababa, Ethiopia

**Keywords:** Neonate, Mortality, Ethiopia, Survival, NICU

## Abstract

**Background:**

Neonatal mortality remains one of the most pressing global health challenges, disproportionately affecting low- and middle-income countries. In Ethiopia, complex interrelated factors contribute to high neonatal mortality rates, creating a multifaceted public health concern that demands comprehensive investigation. Despite the magnitude of this challenge, there exists a notable lack of longitudinal studies that examine the temporal patterns of neonatal deaths, survival, and associated risk factors throughout the complete neonatal period. This study addresses this gap by evaluating survival outcomes and identifying determinants of mortality among neonates admitted to the neonatal intensive care unit at Zewditu Memorial Hospital, a referral facility serving a high-burden urban population in Addis Ababa, Ethiopia.

**Methods:**

A retrospective cohort study of 1,014 neonates admitted to the neonatal intensive care unit from September 11, 2020 to September 10, 2023 was conducted. Systematic random sampling was used to select patients. An electronic data collection method was used, and the data were exported to STATA version 17.0 for cleaning and analysis. Bi-variable and multivariable Cox proportional hazard regression models were employed to identify mortality risk predictors. Kaplan-Meier survival estimates and log-rank tests were used to compare survival probabilities. Model adequacy was assessed using the Cox–Snell residual test.

**Results:**

Over 7,945 neonate-days, the mortality incidence rate was 13.72 deaths per 1,000 neonate-days (95% confidence interval (CI) [11.37–16.55]), with most deaths occurring within the first week. Significant predictors of mortality risk were extremely low birth-weight (<1,000 g; Adjusted Hazard Ratio (AHR) = 18.94, 95% CI [7.89–45.23]), very low birth weight (1,000–1,499 g; AHR = 5.09, 95% CI [2.75–9.43]), perinatal asphyxia (AHR = 3.52, 95% CI [2.25–5.49]), neonatal sepsis (AHR = 2.47, 95% CI [1.28–4.77]), respiratory distress syndrome (AHR = 1.65, 95% CI [1.01–2.68]), and meconium aspiration syndrome (AHR = 2.35, 95% CI [1.20–4.57]).

**Conclusion:**

This study found associations between elevated neonatal mortality rates and birth weight and specific perinatal complications as key factors linked to survival. The predominance of deaths within the first week, particularly among neonates with low birth weight, perinatal asphyxia, sepsis, respiratory distress syndrome, and meconium aspiration syndrome, necessitates the implementation of risk-stratified care and intensive early monitoring. These findings provide evidence for targeted interventions that could substantially reduce neonatal mortality in resource-constrained healthcare settings.

## Introduction

The neonatal period, which is defined as the first 28 days of life, is considered to be the most vulnerable phase for a child’s survival (UNICEF, 2025). Neonatal mortality refers to the death of newborns within this period and is reported as the number of neonatal deaths per 1,000 live births (United Nations Inter-agency Group for Child Mortality Estimation (UN IGME), 2023). Globally, an estimated 2.3 million children died in their first month of life in 2021, averaging 6,400 neonatal deathsdaily, or approximately 18 deaths per 1,000 live births (United Nations Inter-agency Group for Child Mortality Estimation (UN IGME), 2021, United Nations Inter-agency Group for Child Mortality Estimation (UN IGME), 2023; Our World in Data, 2025; UNICEF, 2025). In 2019, approximately one million neonates died worldwide within their first 24 h of life (United Nations Inter-agency Group for Child Mortality Estimation (UN IGME), 2021). Although neonatal mortality has declined globally, the pace of decline has been much slower than expected (UNICEF, 2025). Consequently, the contribution of neonatal deaths to all under-five deaths increased from 40% in 1990 to 47% in 2021 (United Nations Inter-agency Group for Child Mortality Estimation (UN IGME), 2023; UNICEF, 2025).

Sub-Saharan Africa records the highest neonatal mortality globally, with a neonatal mortality rate (NMR) of 27 deaths per 1,000 live births, accounting for 43% of all newborn deaths worldwide (United Nations Inter-agency Group for Child Mortality Estimation (UN IGME), 2023; UNICEF, 2025). Children born in sub-Saharan countries are ten times more likely to die within the first month of life than those born in high-income countries (United Nations Inter-agency Group for Child Mortality Estimation (UN IGME), 2023; UNICEF, 2025).

Child survival is recognized by the international community as a critical marker of a developing society under the Sustainable Development Goals (SDGs). Under SDG 3, Target 3.2, all countries aim to end preventable deaths of newborns, reducing neonatal mortality to at least 12 deaths per 1,000 live births by 2030 (United Nations Inter-agency Group for Child Mortality Estimation (UN IGME), 2023; United Nations Economic Commission for Europe (UNECE), 2025). At the current rate, 63 countries will miss the SDG NMR target (United Nations Inter-agency Group for Child Mortality Estimation (UN IGME), 2023). Even though Ethiopia’s NMR is improving moderately, it remains insufficient to attain the SDG by 2030 (Sachs et al., 2023).

Globally, the contribution of neonatal mortality to overall mortality is substantial and unparalleled by that of any other condition (United Nations Inter-agency Group for Child Mortality Estimation (UN IGME), 2021, United Nations Inter-agency Group for Child Mortality Estimation (UN IGME), 2023). While developed countries maintain very low NMRs, low-income nations and conflict areas of the world experience the highest losses (United Nations Inter-agency Group for Child Mortality Estimation (UN IGME), 2023; Lam et al., 2023). These figures are expected to be exacerbated by the recent COVID-19 pandemic, which caused shortages of basic clinical care and equipment (Roberton et al., 2020). Additionally, the COVID-19 pandemic increased the rate of preterm delivery among infected pregnant women (Blitz et al., 2021; Lin et al., 2023), and prematurity is one of the leading causes of neonatal mortality worldwide (Kedy Koum et al., 2014; Liu et al., 2016; Fottrell et al., 2015). Beyond immediate mortality, neonatal conditions significantly affect survivors, increasing the risk of chronic disorders and disabilities that continue into adulthood (GBD 2019 Diseases and Injuries Collaborators, 2020).

Ethiopia ranks fifth among countries with the highest absolute number of neonatal deaths in the world (World Health Organization & United Nations Children’s Fund, 2019). The 2019 Ethiopian Mini Demographic and Health Survey revealed that the NMR for the five years preceding the survey was 33 deaths per 1,000 live births (Ethiopian Public Health Institute (EPHI), Federal Ministry of Health- FMoH, ICF, 2021). The 2016 Ethiopian Demographic and Health Survey (EDHS) revealed an NMR for the five years preceding the survey of 29 deaths per 1,000 live births (Central Statistical Agency of Ethiopia, ICF, 2016), indicating an upward trend in NMR over recent years. The average number of neonatal deaths in Ethiopia is also higher than the averages for sub-Saharan Africa (27 per 1,000 live births) and southern Asia (22 per 1,000 live births) (UNICEF, 2025).

Approximately 80% of all neonatal deaths worldwide are attributed to three leading causes: prematurity and low birth weight, perinatal complications and asphyxia, and sepsis and infection (Kedy Koum et al., 2014; Liu et al., 2016; Fottrell et al., 2015). These causes vary by region and the timing within the neonatal period (Sankar et al., 2016; United Nations Inter-agency Group for Child Mortality Estimation (UN IGME), 2021; United Nations Inter-agency Group for Child Mortality Estimation (UN IGME), 2023; Rosa-Mangeret et al., 2022). A large prospective cohort study using verbal autopsies in eight countries across sub-Saharan Africa and South Asia revealed that birth asphyxia and sepsis accounted for almost 70% of neonatal deaths, while preterm birth complications remained the third leading causes of neonatal mortality (Alliance for Maternal and Newborn Health Improvement (AMANHI) mortality study group, 2018).

In Ethiopia, neonatal mortality remains a significant challenge despite government-led interventions and partnerships (Federal Ministry of Health of Ethiopia, 2017). Nevertheless, these efforts have fallen short of achieving national and global targets, including SDG 3.2 (Sachs et al., 2023). Consequently, neonatal mortality remains a critical area of research requiring further intervention. There are limited data on the survival status and predictors of neonatal mortality in Ethiopia, particularly within the current study area. Additionally, there is a paucity of research with follow-up periods spanning over the entire neonatal period. Thus, this study aimed to identify the survival status and predictors of mortality among neonates admitted to the neonatal intensive care unit (NICU) of Zewditu Memorial Hospital (ZMH) and to identify the critical time for targeted interventions.

## Materials & Methods

### Study setting

This study was conducted at the NICU of ZMH, a referral hospital in Addis Ababa, Ethiopia. This hospital is renowned for its pediatric services which include pediatric outpatient services including sub-specialty clinics, pediatric emergency services, pediatric in-patient services, a stabilization center for severe acute malnutrition patients, NICU and vaccination services. The NICU has a capacity of 40 beds, with an average of 1,200 neonates receiving inpatient care annually. The care team comprises17 BSc nurses,eight neonatal nurses, three general practitioners, one pediatrician and one neonatologist providing care within the NICU.

### Study design

A hospital-based retrospective cohort study design was employed. The study was reported in accordance with the Strengthening the Reporting of Observational Studies in Epidemiology (STROBE) guidelines.

### Source population

The source population included all live neonates admitted to the NICU of ZMH between September 11, 2020 and September 10, 2023.

### Sample population

The sample population consisted of neonates whose charts were selected using the specified sampling technique.

### Exclusion criteria

Neonates with missing medical records were excluded

### Sample size determination and sampling technique

**Figure 1:**
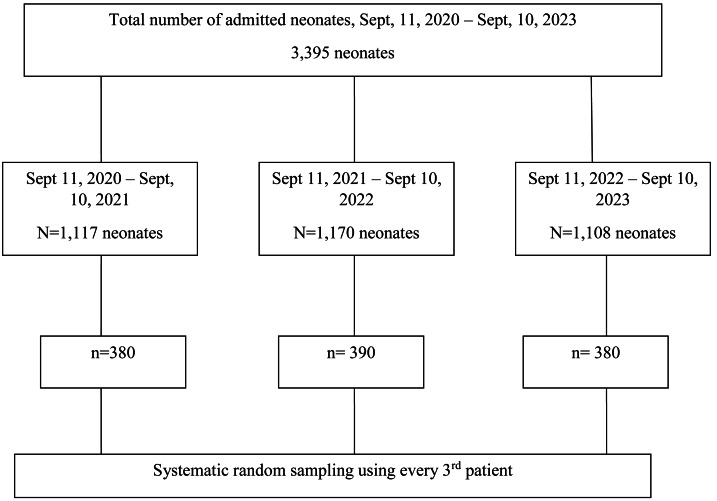
Schematic presentation of sampling procedure to assess time to death and predictors of time to death among neonates admitted to the NICU of ZMH from Sept 11, 2020–Sept 10, 2023.

To determine predictors of neonatal mortality risk, the sample size was calculated using the Cox method, with variability of 0.5, a 95% confidence interval, a 5% margin of error, and a 5% contingency. A failure probability of 0.0192 was sourced from a previous study conducted at Tikur Anbessa Hospital (Menalu et al., 2022). The final calculated sample size for this study was 1,150. Of these, 136 charts (11.8%) were missing and therefore excluded from the study. Consequently, a total of 1,014 neonatal medical records (88.2%) were included in the final analysis. A systematic random sampling technique was used to select medical record numbers of the charts that were included in the study. The sample was proportionally allocated to each year. The total number of neonates admitted to the NICU (N) was divided by the sample size (n) to yield a sampling interval of *k* = 3. Accordingly, every third chart on the Health Management Information System (HMIS) register was selected (Fig. 1).

### Operational definitions

Event: Death following NICU admission.

Time to death: The duration from NICU admission to the occurrence of the outcome

Censoring: Neonates were censored if they were discharged home (recovered), discharged against medical advice, referred to other health institutions with an unknown outcome, or remained in the NICU beyond 28 days of age.

### Data collection and data quality assurance

A data abstraction tool was developed following a thorough literature review of similar studies. The tool was constructed in English and applied without translation to local languages as English is the standard language for medical records in the study setting. Data were collected electronically. To ensure data quality, the tool was pretested on 5% of the sample size at Gandhi Memorial Hospital prior to data collection. The tool was refined based on the pretest findings, such as the addition of specific maternal and neonatal diagnoses. Data were collected by four medical doctors from different departments at ZMH with prior experience in electronic data collection. These individuals underwent a half-day training session on the tool’s content and extraction procedures. Patient information was extracted from medical records and entered into an electronic data extraction form by the trained collectors, with daily supervision provided by the principal investigator

### Data processing and analysis

Electronically collected data were exported to Microsoft Excel and then to STATA 17 statistical software for cleaning and analysis. A complete case analysis was performed for all included patients. Missing data were <5% for all key variables and assumed to be missing randomly. All predictors were assessed at NICU admission, prior to the start of follow-up. Continuous data were described using mean and standard deviation and median and interquartile range (IQR). Categorical data were presented using frequency distributions. The outcome of each neonate was dichotomized into censored or death. The mortality incidence rate was calculated by dividing the number of deaths by the total neonate-days at risk and reported per 1,000 neonate-days. The Kaplan–Meier method was used to estimate survival time and cumulative probability of survival, and the log-rank test was used to compare survival curves. A Cox proportional hazard regression model was used to analyze the relationship between predictors and mortality risk. Bivariable analysis was performed for all variables; those with a *P*-value ≤ 0.25 were included in the multivariable analysis. Before multivariable analysis, multicollinearity was assessed using the Variance Inflation Factor (VIF), and the mean VIF obtained was 1.40, indicating no multicollinearity. In the multivariable analysis, a *P*-value < 0.05 was used to declare statistically significant associations. Associations were summarized using adjusted hazard ratios (AHR) at 95% confidence intervals (CI). The proportional hazard assumption test yielded a global test *p*-value of 0.854, signifying that the assumption was met. Finally, the results were presented using text, tables, and graphs.

### Ethical approval and consent to participate

Ethical clearance was obtained from the institutional review boards of Addis Ababa Public Health Research and Emergency Management Directorate (Ref AAH/9589/227) and Addis Continental Institute of Public Health. The study utilized secondary data obtained from patient medical records of neonates admitted to the NICU. No direct patient contact occurred, and therefore informed consent from parents or guardians was waived. Permission for data access was secured from the ZMH administration. To protect patient privacy, all personally identifiable information was removed upon data extraction, and all analyses were performed on a fully anonymized dataset.

## Results

### Socio-demographic characteristics of neonates and their mothers

Of the 1,014 study participants, 579 (57.10%) were male. A total of 709 (69.92%) neonates were admitted to the NICU within the first 24 h of life. The median neonatal age at admission was 1 day (IQR: 1 to 2 days). The majority of the mothers 750 (78.45%) who gave birth to the neonates belonged to the age group of 21-34 years. The documented maternal age ranged from 17 to 45 years, with a median age of 26 (IQR: 23–30) (Table 1).

**Table 1:** Socio-demographic characteristics of neonate and mother in NICU, ZMH, Addis Ababa, Ethiopia, from Sept 11, 2020–Sept 10, 2023.

	Category	Total n (%)	Outcome		Log rank test	
			Censored Count (%)	Died Count (%)	X^2^	*P*-value
Sex of neonate	Male	579 (57.10)	514 (88.77)	65 (11.23)	1.03	0.310
	Female	435 (42.90)	391 (89.89)	44 (10.11)		
Maternal age	≤20	98 (10.25)	85 (86.73)	13 (13.27)	1.47	0.688
	21–34	750 (78.45)	671 (89.47)	79 (10.53)		
	35–45	105 (10.98)	96 (91.43)	9 (8.57)		
	≥45	3 (0.31)	3 (100)	0		
Neonatal age at admission	0–1 day	709 (69.92)	616 (86.88)	93 (13.12)	14.73	0.002
	2–7 days	202 (19.92)	191 (94.55)	11 (5.45)		
	8–14 days	57 (5.62)	52 (91.23)	5 (8.77)		
	15–28 days	46 (4.54)	46 (100)	0		

### Maternal obstetric history

In this study, 584 (57.59%) of the mothers were primiparous and 929 (91.62%) attended at least one ante natal care (ANC) follow up. Regarding the order of the index pregnancy, 905 (89.25%) neonates were singleton. Of the total enrolled, 648 (63.91%) neonates were born at ZMH and more than half were delivered *via* spontaneous vaginal delivery (SVD) (Table 2).

**Table 2:** Maternal obstetric history and other health related conditions in NICU, ZMH, Addis Ababa, Ethiopia, from Sept 11, 2020–Sept 10, 2023.

Variables	Category	**Total**	Outcome		Log rank test	
			Censored	Died	X^2^	*P* value
Parity	Primipara	584 (57.59)	523 (89.55)	61 (10.45)	0.06	0.969
	Multipara	418 (41.22)	372 (89.0)	46 (11.0)		
	Unspecified	12 (1.18)	10 (83.33)	2 (16.67)		
Antenatal care	Yes	929 (91.62)	832 (89.56)	97 (10.44)	1.23	0.539
	No	19 (1.87)	16 (84.21)	3 (15.79)		
	Unspecified	66 (6.51)	57 (86.36)	9 (13.64)		
Order of current pregnancy	Singleton	905 (89.25)	815 (90.06)	90 (9.94)	1.27	0.531
	Twin	102 (10.06)	85 (83.33)	17 (16.67)		
	Triplet	7 (0.69)	5 (71.43)	2 (28.57)		
Mode of current delivery	Spontaneous vaginal	568 (56.02)	503 (88.56)	65 (11.44)	1.33	0.723
	Assisted vaginal delivery	51 (5.03)	48 (94.12)	3 (5.88)		
	Cesarean section	392 (38.66)	351 (89.54)	41 (10.46)		
	unspecified	3 (0.30)	3 (100)	0 (0)		
Place of delivery	ZMH	648 (63.91)	574 (88.58)	74 (11.42)	0.89	0.347
	Others	366 (36.09)	331 (90.44)	35 (9.56)		

### Maternal obstetric and medical complications

Among the 276 (27.22) mothers with documented illness, 231 (83.70%) experienced obstetric complications. Of these, 38 (16.45%) presented with pre-eclampsia, among whom eight (21.05%) neonates died. Additionally, 60 (25.97%) had premature rupture of membranes (PROM), 19 (8.23%) had antepartum hemorrhage (APH) and 34 (14.72%) had previous cesarean section scars. Among the mothers with documented illness, 64 (23.19%) presented with medical complications. Of these mothers, 25 (39.06%) had HIV, out of whom four (16.00%) of their neonates died. Hypertension and diabetes were also documented in seven (10.94%) and 18 (28.13%) mothers, respectively (Table 3).

**Table 3:** Maternal obstetric and medical conditions at NICU, ZMH, Addis Ababa, Ethiopia, from Sept 11, 2020–Sept 10, 2023.

Variables	Categories	Total	Outcome		Log rank test	
			Censored	Died	X^2^	*P* value
Obstetric *n* = 231						
Pre-eclampsia	Yes	38 (16.45)	30 (78.95)	8 (21.05)	2.91	0.088
	No	193 (83.55)	175 (90.67)	18 (9.33)		
Premature rupture of membranes (PROM)	Yes	60 (25.97)	57 (95.0)	3 (5.0)	1.44	0.230
	No	171 (74.03)	148 (86.55)	23 (13.45)		
Previous cesarean section (C/S)	Yes	34 (14.72)	32 (94.12)	2 (5.88)	0.84	0.359
	No	197 (85.28)	173 (87.82)	24 (12.18)		
Ante partum hemorrhage (APH)	Yes	19 (8.23)	16 (84.21)	3 (15.79)	0.16	0.689
	No	212 (91.77)	189 (89.15)	23 (10.85)		
	No	214 (92.64)	188 (87.85)	26 (12.15)		
Medical *n* = 64						
Hypertension	Yes	7 (10.94)	6 (85.71)	1 (14.29)	0.01	0.928
	No	57 (89.06)	50 (87.72)	7 (12.28)		
Human immunodeficiency virus (HIV)	Yes	25 (39.06)	21 (84)	4 (16.0)	0.47	0.495
	No	39 (60.94)	35 (89.74)	4 (10.26)		
Diabetes mellitus	Yes	18 (28.13)	16 (88.86)	2 (11.11)	0.04	0.847
	No	46 (71.88)	40 (86.96)	6 (13.04)		
Heptitis B virus (HBV)	Yes	5 (7.81)	4 (80.0)	1 (20.0)	0.35	0.551
	No	59 (92.19)	52 (88.14)	7 (11.86)		

### Neonatal medical conditions

In this study, 298 (29.39%) of the neonates were born prematurely; among these, 61 (20.47%) died. The mean birth weight was 2,670.38 grams ± 831.03 grams. Ten (1.05%) neonates weighed <1,000 grams at birth, among whom only one survived. Of the 84 (8.84%) neonates with birth weight between 1,000–1,499 grams, 36 (42.86%) died. Close to two-thirds of the neonates had temperature recordings below 36.5 °C at the time of admission. The median temperature at admission was 36.1 °C (IQR: of 35.2 °C–36.8 °C). The minimum recorded temperature was 30.9 °C and the maximum was 41 °C.

The most frequent medical problems documented among neonates were neonatal sepsis 719 (70.91), respiratory distress syndrome (RDS) 223 (21.99%), perinatal asphyxia (PNA) 130 (12.82%), and neonatal jaundice 267 (26.33%). The other medical conditions are listed in the table below (Table 4). Higher mortality was recorded among neonates with PNA (24.62%), RDS (25.56%) and neonatal sepsis (13.63%) (Table 4).

**Table 4:** Medical conditions of neonates who were admitted at NICU of ZMH, Addis Ababa, Ethiopia, from Sept 11, 2020 –Sept 10, 2023.

Variables	Categories	Total n (%)	Outcome		Log rank test	
			Censored	Died	X^2^	*P* value
Maturity of the neonate	AGA	660 (65.09)	586 (88.79)	74 (11.21)	4.83	0.184
	LGA	46 (4.54)	42 (91.30)	4 (8.70)		
	SGA	125 (12.33)	107 (85.60)	18 (14.40)		
	Unknown	183 (18.05)	170 (92.90)	13 (7.10)		
Gestational age	Preterm	298 (29.39)	237 (79.53)	61 (20.47)	18.70	0.000
	Term	500 (49.31)	469 (93.80)	31 (6.20)		
	Post term	50 (4.93)	46 (92.0)	4 (8.0)		
	Unknown	166 (16.37)	153 (92.17)	13 (7.83)		
Birth weight	<1,000	10 (1.05)	1 (10.0)	9 (90.0)	128.18	0.000
	1,000–1,499	84 (8.84)	48 (57.14)	36 (42.86)		
	1,500–2,499	249 (26.21)	224 (89.96)	25 (10.04)		
	2,500–3,999	558 (58.74)	526 (94.27)	32 (5.73)		
	≥4,000	49 (5.16)	47 (95.92)	2 (4.08)		
Admission temperature	<36.5	633 (62.74)	552 (87.20)	81 (12.80)	7.81	0.020
	36.5–37.4	265 (26.26)	249 (93.96)	16 (6.04)		
	≥37.5	111 (11.0)	100 (90.09)	11 (9.91)		
Myelomeningocele	Yes	93 (9.17)	86 (92.47)	7 (7.53)	2.85	0.092
	No	921 (90.83)	819 (88.93)	102 (11.07)		
Perinatal asphyxia	Yes	130 (12.82)	98 (75.38)	32 (24.62)	27.35	0.000
	No	884 (87.18)	807 (91.29)	77 (8.71)		
Respiratory distress syndrome	Yes	223 (21.99)	166 (74.44)	57 (25.56)	35.39	0.000
	No	791 (78.01)	739 (93.43)	52 (6.57)		
Necrotizing enterocolitis	Yes	31 (3.06)	19 (61.29)	12 (38.71)	8.89	0.003
	No	983 (96.94)	886 (90.13)	97 (9.87)		
Neonatal jaundice	Yes	267 (26.33)	254 (95.13)	13 (4.87)	15.22	0.000
	No	747 (73.67)	651 (87.15)	96 (12.85)		
Neonatal sepsis	Yes	719 (70.91)	621 (86.37)	98 (13.63)	14.75	0.000
	No	295 (29.09)	284 (96.27)	11 (3.73)		
Meconium aspiration syndrome	Yes	98 (9.66)	85 (86.73)	13 (13.27)	1.87	0.171
	No	916 (90.34)	820 (89.52)	96 (10.48)		
Apnea of prematurity	Yes	11 (1.08)	6 (54.55)	5 (45.45)	7.01	0.008
	No	1,003 (98.92)	899 (89.63)	104 (10.37)		
Type of feeding	Breast milk	623 (87.62)	594 (95.35)	29 (4.65)	11.72	0.008
	Formula milk	34 (4.78)	31 (91.18)	3 (8.82)		
	Mixed feeding	20 (2.81)	20 (100.0)	0 (0)		
	Unspecified	34 (4.78)	29 (85.29)	5 (14.71)		

### Survival status of neonates

A total of 1,014 neonates who were admitted to the NICU during the study period were followed from 1 to 28 days of age. The median duration of hospital stay was 5 days (IQR: 3–11 days). The minimum and maximum follow up times for this study were 1 and 28 days, respectively. During the follow-up period, 109 (10.75%) of the study participants died. Out of the 109 deaths, 91 (83.49%) occurred within the first seven days of follow-up, 10 (9.17%) occurred between days 8 and 14, and the remaining eight (7.34%) occurred between days 15 and 28. The cumulative probability of death at the end of the 7th, 14th and 28th days was 0.12, 0.15 and 0.23, respectively. Censorship was recorded for 905 (89.25%) neonates, of which 776 (85.75%) were discharged, 21 (2.32%) left against medical advice, 22 (2.43%) were referred out to other health facilities with unknown outcomes and 86 (9.50%) the neonates stayed beyond 28 days of life.

### Failure of neonates and overall Kaplan–Meier survival function

The total follow-up in this study was 7,945 neonate-days, with an overall neonatal mortality incidence rate of 13.72 deaths per 1,000 neonate day observations (95% CI [11.37–16.55]). Neonates admitted on the first day of life exhibited a higher mortality incidence rate of 16.60 deaths per 1,000 neonate-days (95% CI [13.54–20.34]). The mortality incidence rate was also higher among neonates born prematurely (18.66; 95% CI [14.52–23.98]) compared to those born at term (10.26; 95% CI [7.22–14.60]). Similarly, extremely low birth weight (ELBW) and very low birth weight (VLBW) neonates had high mortality incidence rates of 152.54 (95% CI [79.37–293.17]) and 32.64 (95% CI [23.54–45.25]) per 1,000 neonate-days respectively. Low birth weight (LBW) and normal birth weight (NBW) neonates had incidence rates of death of 10.07 (95% CI [6.80–14.90]) per 1,000 and 9.46 (95% CI [6.69–13.38]) per 1,000 neonatal days respectively.

**Figure 2:**
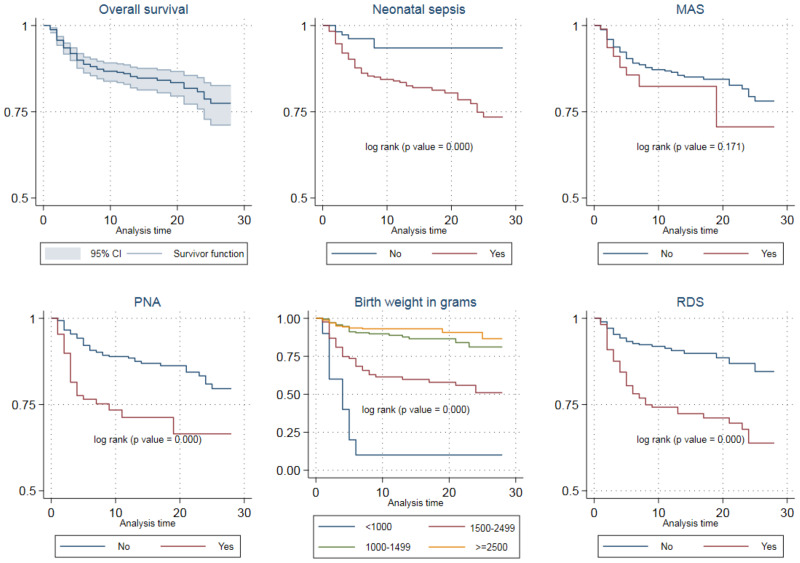
Overall Kaplan Meier Survival estimate and survival estimates by each predictor variables of neonates who were admitted at NICU of ZMH, Addis Ababa, Ethiopia, from Sept 11, 2020–Sept 10, 2023.

On the first day of hospital stay, the neonatal mortality incidence rate was11.85 deaths (95% CI [6.72–20.86]) per 1,000 neonate follow-up days. The highest mortality incidence rate occurred during the first week of hospital stay, at 21.14 deaths (95% CI [16.96–26.36]) per 1,000 neonate-days, while the lowest occurred in the third week at 4.13 deaths (95% CI [1.62–11.47]) per 1,000 neonate-days. As shown in the overall Kaplan–Meier survival curve (Fig. 2), neonates had the highest survival on the first day of follow up; and their chances of survival decreased as the duration of follow up increased. A steeper decrease in survival was observed in the first five days of admission. Survival also decreased over time in the NICU for each of the predictor variables (Fig. 2).

### Predictors of time to death among neonates admitted to NICU

**Figure 3:**
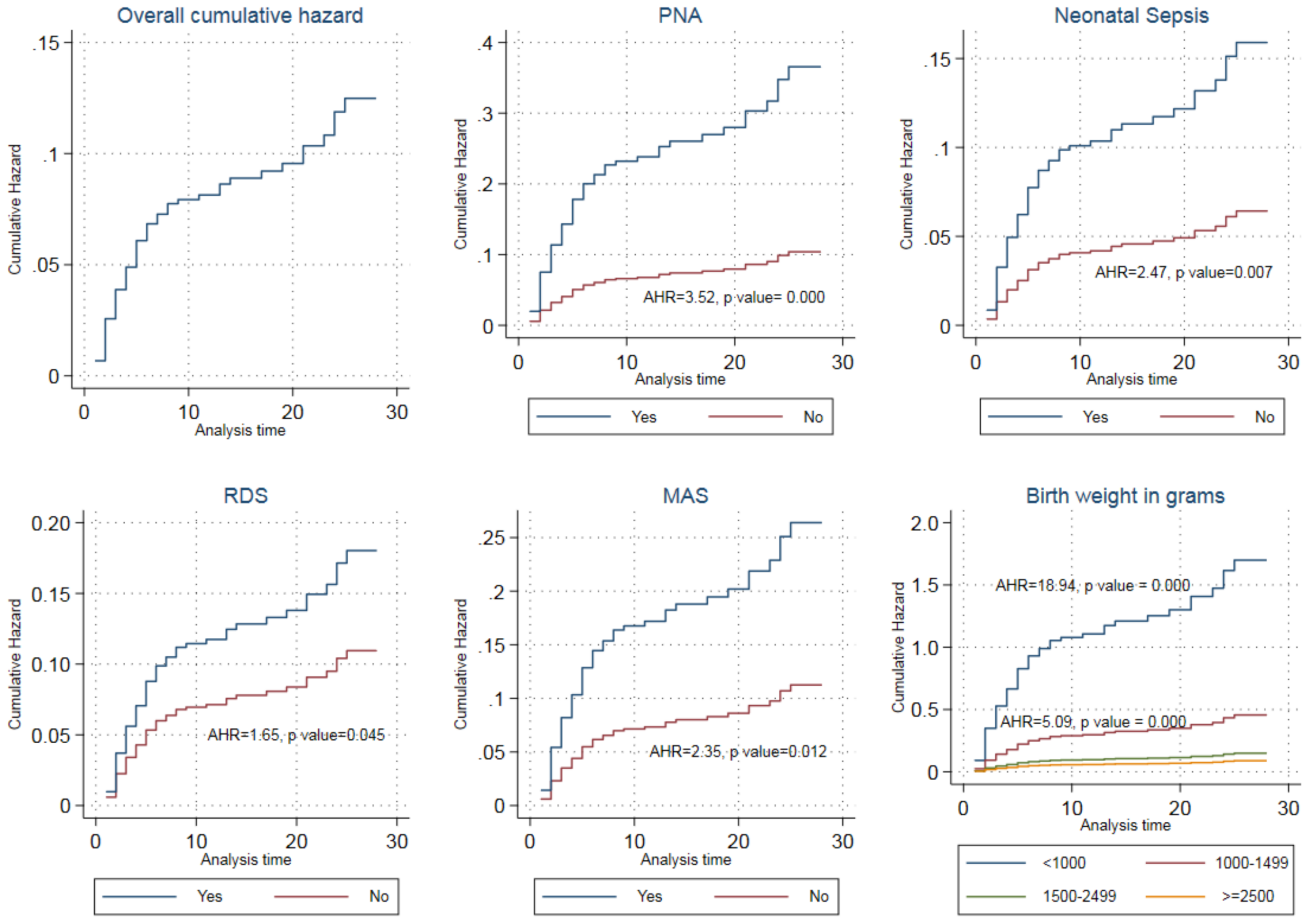
Overall cumulative hazard and hazard of the predictors of neonatal mortality among neonates admitted at NICU of ZMH, Addis Ababa, Ethiopia, from Sept 11, 2020–Sept 10, 2023.

Multivariable Cox regression analysis identified several independent predictors of neonatal mortality risk: birth weight less than 1,000 grams (AHR = 18.94, 95% CI [7.89–45.23]), birth weight between 1,000 –1,499 grams (AHR = 5.09, 95% CI [2.75–9.43]), PNA (AHR = 3.52, 95%CI [2.25–5.49]), neonatal sepsis (AHR = 2.47, 95% CI [1.28–4.77]), meconium aspiration syndrome (MAS) (AHR = 2.35, 95% CI [1.20–4.57]), and RDS (AHR = 1.65, 95% CI [1.01–2.68]). Neonates born with a birth weight <1,000 grams had an 18.94-fold higher hazard of death than those with a birth weight ≥ 2,500 grams. Similarly, neonates born with a birth weight between 1,000–1,499 grams had a 5.09-fold higher hazard of death than those with a birth weight ≥ 2,500 grams. Neonates with neonatal sepsis had a 2.47-fold higher hazard of mortality compared to those without sepsis. Neonates with PNA had a 3.25-fold higher hazard of mortality compared to those without PNA. Furthermore, neonates diagnosed with MAS and RDS had a 2.35-fold and 1.65-fold higher hazard of mortality, respectively, than neonates without these conditions. (Table 5) (Fig. 3)

**Table 5:** Bivariable and multivariable Cox regression on predictors of neonatal mortality of neonates who were admitted at NICU of ZMH, Addis Ababa, Ethiopia, from Sept 11, 2020–Sept 10, 2023.

Variables	Categories	N (%)	CHR (95% CI)	AHR (95% CI)
Birth weight in grams	<1,000	10(1.05)	17.01 (8.15–35.50)	18.94 (7.89–45.23)
	1,000–1,499	84(8.84)	5.05 (3.13–8.16)	5.09 (2.75–9.43)
	1,500–2,499	249 (26.21)	1.36 (0.81–2.29)	1.67 (0.93–3.00)
	≥2,500	558 (58.74)	1	1
Neonatal sepsis	No	295 (29.09)	1	1
	Yes	719 (70.19)	3.15 (1.69–5.88)	2.47 (1.28–4.77)
Perinatal asphyxia	No	884 (87.18)	1	1
	Yes	130 (12.82)	2.84 (1.88–4.29)	3.52(2.25-5.49)
Meconium aspiration syndrome	No	916 (90.34)	1	1
	Yes	98 (9.66)	1.49 (0.83–2.64)	2.35 (1.20–4.57)
Respiratory distress syndrome	No	791 (78.01)	1	1
	Yes	223 (21.99)	2.98 (2.04–4.36)	1.65 (1.01–2.68)

## Discussion

This study revealed that the major neonatal diagnoses observed were neonatal sepsis, neonatal jaundice, RDS, PNA, and MAS. Similar patterns of neonatal diagnoses have been observed in different studies conducted in different parts of Ethiopia (Dessu et al., 2020; Eyeberu et al., 2021; Tolossa et al., 2022; Fentaw & Demis, 2024). Our study showed that the overall incidence of neonatal mortality was 13.72 per 1,000 neonates-days. This finding is in line with a previous study conducted in Tikur Anbessa Specialized Hospital (11 per 100 admitted neonates) (Menalu et al., 2022). The incidence reported in this study is higher than that reported in a study conducted in Southern Ethiopia in the Aroresa district, which reported 4.1 deaths per 100 admissions (Limaso, Dangisso & Hibstu, 2020). This difference in neonatal mortality can be explained by the fact that the district hospital in Aroresa is at a lower tier than ZMH, managing less severe cases and referring out difficult patients. Studies conducted in Northern Ethiopia, in the Amhara region, reported higher neonatal mortality rates, with 30.6 and 17.3 deaths per 100 admissions in Gondar and Bahirdar, respectively (Eyeberu et al., 2021; Yeshaneh et al., 2021). These findings of higher neonatal mortality can be attributed to the fact that these comprehensive and specialized hospitals deal with complicated cases referred from lower-tier hospitals. This can also be because these hospitals have predominantly rural catchment areas where healthcare seeking behavior and accessibility are limited as compared to urban areas. The variations in mortality rates may be also be attributed to several other methodological differences in study settings, sample size, study duration, and in the socio-demographic characteristics of the study participants.

This study revealed a steep decrease in the probability of neonatal survival during the first seven consecutive follow-up days. Similar findings were evidenced by studies conducted in different parts of Ethiopia. A study conducted in the NICU of Woldia Hospital showed that 63.24% of neonatal deaths occurred in the first seven days of life (Fentaw & Demis, 2024). The proportion of neonates who died in the first week of life were 70% in Tikur Anbessa Specialized Hospital (Menalu et al., 2022) and 83.5% in Debre Markos Referral Hospital (Alebel et al., 2020). These consistent findings indicate that the first week of admission to the NICU is the most critical time. This might be explained by the impact of not only neonatal conditions but also poorly identified and managed intrapartum conditions (Tesfay et al., 2022).

This study has shown that as birth weight decreases below 2,500 grams, survival chances also decrease. Other studies have reported similar findings. A study conducted in the NICU of a tertiary center in India showed that ELBW (adjusted odds ration (AOR) = 25.11, 95% CI [5.71–110.24]) and VLBW (AOR = 4.75, 95% CI [2.21–10.32]) deliveries were significant predictors of mortality(Singh et al., 2023). Studies that assessed predictors of neonatal mortality in Ethiopia did not classify low birth weight as very low or extremely low but rather reported it as low birth weight. Similar to this study, LBW is a predictor of mortality in multiple studies, such as that conducted in Bombe Primary Hospital, with an AHR = 2.59 (95% CI [1.1–6.26]) (Berhanu et al., 2021), Tikur Anbessa Specialized Hospital, with an AHR = 7.3 (95% CI [2.69–19.91]) (Menalu et al., 2022), and Hiwot Fana Specialized University Hospital, with an AHR = 4.01 (95% CI [1.30–12.33]) (Eyeberu et al., 2021). Although neonates with a birth weight <1,000g had a significantly higher hazard of mortality (AHR = 18.94; 95% CI [7.89–45.23]), the wide confidence interval reflects substantial uncertainty, and future studies with larger sample sizes are needed to validate this finding.

PNA was identified as another predictor of neonatal mortality in this study. This finding aligns with studies conducted in Ethiopia and internationally. A study conducted in the NICU of Woldia Hospital reported an AHR for PNA to be 1.64 (95% CI [1.05–2.58]) (Fentaw & Demis, 2024). Other studies, such as those conducted in Tikur Anbessa Specialized Hospital (AHR = 5.2, 95% CI [1.92–14.30]) (Menalu et al., 2022), Hiwot Fana Specialized University Hospital (AHR = 3.85, 95% CI [1.83–8.10]), and a referral hospital in Cameroon (AOR = 16.4, 95% CI [6.35–42.47]) showed a stronger association between PNA and neonatal mortality. This finding may correlate with neonatal care given not only in the NICU but also the effectiveness of resuscitation efforts in the delivery room.

Neonatal sepsis was another predictor of mortality identified in this study, consistent with findings from other studies on neonatal mortality predictors. A study at Woldia Hospital found that neonates with sepsis had a 1.87-fold higher hazard of death (AHR = 1.87, 95% CI [1.23–2.86]) (Fentaw & Demis, 2024). Other similar studies conducted in Tikur Anbessa Specialized Hospital (AHR = 3.4, 95% CI [1.71–4.01]) (Menalu et al., 2022) and Hiwot Fana Specialized University Hospital (AHR=3.93, 95% CI [1.84–8.41]) also identified sepsis as a predictor of neonatal mortality.

RDS as a predictor of neonatal mortality has also been reported in other similar studies, such as those conducted in Tikur Anbessa Specialized Hospital (AHR = 2.5, 95% CI [1.24–5.09]) and in Debre Markos Referral Hospital (AHR = 2, 95% CI [1.3–3.1]). This association may be partly due to the limited availability of neonatal mechanical ventilators and surfactants, which are critical for treating RDS, in most NICUs across Ethiopia, including ZMH.

The findings from this research can guide improvements in the quality of neonatal care at ZMH and other similar facilities. Furthermore, they may serve as a foundation for further investigation into factors influencing neonatal outcomes and the overall standard of care in hospital settings.

### Limitations

This study has some limitations. First, it relied on neonatal medical records as the primary source of data, which are inadequate for capturing maternal information and lack means of triangulation. Second, the study is susceptible to temporal bias because of its retrospective chart review design, which limits the ability to account for changes in NICU practices and protocols over time. Third, there were unmeasured confounders, such as maternal socioeconomic status, educational level, and marital status, which may have influenced the study outcomes. Although we adjusted for several known confounders in our analysis, unmeasured factors like socioeconomic status and maternal education could contribute to residual confounding. These factors might independently affect neonatal outcomes, potentially biasing our results.

Additionally, the exclusion of 136 charts because of loss may have introduced selection bias and impacted the representativeness of the sample. Although missingness was minimal and assumed to be random, we acknowledge that the excluded charts could have differed systematically from the included records in ways not captured by our analysis. This limitation highlights the need for caution in generalizing the findings to broader populations.

## Conclusions

Based on the findings of this study, neonatal mortality remains high in Ethiopia. Because the majority of the deaths were recorded within the first seven days of follow-up, we recommend that due attention be given to neonates during the early admission days. We also recommend that focused care should be given to neonates admitted with the diagnoses of ELBW, VLBW, MAS, RDS, PNA and neonatal sepsis.

## Supplemental Information

10.7717/peerj.21473/supp-1 Supplemental Information 1 Raw Data

10.7717/peerj.21473/supp-2 Supplemental Information 2 Code Book

10.7717/peerj.21473/supp-3 Supplemental Information 3 STROBE checklist

## Additional Information and Declarations

### Data Availability

The following information was supplied regarding data availability: The raw measurements are available in the Supplemental File.
