# Supplementary material for: Survival and predictors of neonatal mortality: a hospital-based retrospective follow-up study from Addis Ababa, Ethiopia
Source: PeerJ. 2026 Jul 22;14:e21473. doi: 10.7717/peerj.21473 (PMC13401358; doi:10.7717/peerj.21473)
Supplement: Supplemental Information 2 [file peerj-14-21473-s002.docx]

Code Book

| Number | Variables | Coding Categories |
| --- | --- | --- |
|  | Year of admission in Ethiopian calendar |  |
| 1 | Maternal age |  |
| 2 | Mother attended ANC follow up | No = 0  Yes = 1 |
| 2.1 | If yes, number of visits | 1 visit = 1  2 visits = 2  3 visits = 3  ≥4 visits = 4  Unspecified 5 |
| 3 | Mother tested for HIV | No = 0  Yes = 1  Unspecified = 2 |
| 3.1 | If yes to HIV test | Negative = 0  Positive = 1  Unspecified = 2 |
| 4 | History of previous pregnancies |  |
| 4.1 | Primipara | No = 0  Yes = 1 |
| 4.2 | Multipara | No = 0  Yes = 1 |
| 4.3 | Still birth | No = 0  Yes = 1 |
| 4.4 | Abortion | No = 0  Yes = 1 |
| 4.5 | Unspecified | No = 0  Yes = 1 |
| 5 | Order of current pregnancy | Singleton  Twin |
| 6 | Presence of maternal illness | Yes  No |
| 6.1 | If yes to maternal illness | Obstetric  Medical |
| 6.2 | If yes to maternal illness and obstetric |  |
| 6.2.1 | Abortion | No = 0  Yes = 1 |
| 6.2.2 | PROM | No = 0  Yes = 1 |
| 6.2.3 | Prolonged labor | No = 0  Yes = 1 |
| 6.2.4 | Previous C/S | No = 0  Yes = 1 |
| 6.2.5 | Ante partum hemorrhage | No = 0  Yes = 1 |
| 6.2.6 | Oligohydramnios | No = 0  Yes = 1 |
| 6.2.7 | Chorioamnionitis | No = 0  Yes = 1 |
| 6.2.8 | Cephalopelvic disproprtion | No = 0  Yes = 1 |
| 6.2.9 | Multiple pregnancy | No = 0  Yes = 1 |
| 6.2.10 | Preeclampsia | No = 0  Yes = 1 |
| 6.2.11 | Pregnancy related hypertension | No = 0  Yes = 1 |
| 6.2.12 | Prolonged labor | No = 0  Yes = 1 |
| 6.2.13 | Chord prolapse | No = 0  Yes = 1 |
| 6.2.14 | Others | No = 0  Yes = 1 |
| 6.3 | If yes to maternal illness and medical |  |
| 6.3.1 | Hypertension | No = 0  Yes = 1 |
| 6.3.2 | HIV | No = 0  Yes = 1 |
| 6.3.3 | Diabetes | No = 0  Yes = 1 |
| 6.3.4 | Cardiac illness | No = 0  Yes = 1 |
| 6.3.4 | Anemia | No = 0  Yes = 1 |
| 6.3.5 | Chronic lung disease | No = 0  Yes = 1 |
| 6.3.6 | Seizure disoder | No = 0  Yes = 1 |
| 6.3.7 | HBV | No = 0  Yes = 1 |
| 6.3.8 | Others | No = 0  Yes = 1 |
|  | Specify Others |  |
| 7 | Onset of labor | Spontaneous  Induces  Elective C/S  Unspecified |
| 8 | Mode of delivery | SVD  C/S  Assissted vaginal  Unspecified |
| 9 | Duration of labor in hours |  |
| 10 | Place of delivery | ZMH  Other than ZMH |
| 10.1 | If other than ZMH, | In Addis Ababa  Out of Addis Ababa |
| 10.2 | If other than ZMH, | General Hospital  Primary Hospital  Health Center  Health post  Private Institution  Ambulance  Home  Unspecified |
| 11 | Sex of the neonate | Male  Female |
| 12 | Post natal age at admission in days |  |
| 13 | Gestational age | Known  Unknown |
| 14 | Gestational age in weeks |  |
| 15 | Gest_new | Preterm  Term  Post term |
| 16 | Birth weight in grams |  |
| 17 | Birth weight Vs Gestational age on Lubchenco Curve | SGA  AGA  LGA |
| 18 | Reason for referral |  |
| 18.1 | Perinatal asphyxia | No = 0  Yes = 1 |
| 18.2 | Hypothermia | No = 0  Yes = 1 |
| 18.3 | Respiratory distress | No = 0  Yes = 1 |
| 18.4 | Failure to suck | No = 0  Yes = 1 |
| 18.5 | Birth injury | No = 0  Yes = 1 |
| 18.6 | Baby of mother with cardiac illness | No = 0  Yes = 1 |
| 18.7 | Baby of pre eclamptic mother | No = 0  Yes = 1 |
| 18.8 | Baby of PROM mother | No = 0  Yes = 1 |
| 18.9 | HIV exposed neonate | No = 0  Yes = 1 |
| 18.10 | Meconium aspiration syndrome | No = 0  Yes = 1 |
| 18.11 | Jaundice | No = 0  Yes = 1 |
| 18.12 | Prematurity | No = 0  Yes = 1 |
| 18.13 | Low birth weight | No = 0  Yes = 1 |
| 18.14 | VLBW | No = 0  Yes = 1 |
| 18.15 | MMC | No = 0  Yes = 1 |
| 18.16 | Cough | No = 0  Yes = 1 |
| 18.17 | Fever | No = 0  Yes = 1 |
| 18.18 | Convulsions | No = 0  Yes = 1 |
| 18.19 | Others | No = 0  Yes = 1 |
|  | If others, specify |  |
| 19 | Measurement of RBS | Done  Not done |
| 19.1 | If done, value of first RBS measurement in mg/dl |  |
| 20 | Admission temperature in 0C |  |
| 21 | Diagnoses of the neonate |  |
| 21.1 | Neonatal sepsis | No = 0  Yes = 1 |
| 21.2 | Respiratory distress syndrome | No = 0  Yes = 1 |
| 21.3 | Necrotizing enterocolitis | No = 0  Yes = 1 |
| 21.4 | Neonatal jaundice | No = 0  Yes = 1 |
| 21.5 | Apnea of prematurity | No = 0  Yes = 1 |
| 21.6 | Intraventricular hemorrhage | No = 0  Yes = 1 |
| 21.7 | Patent ductus arteriosus | No = 0  Yes = 1 |
| 21.8 | Hospital acquired infection | No = 0  Yes = 1 |
| 21.9 | Meconium aspiration syndrome | No = 0  Yes = 1 |
| 21.10 | Hypothermia | No = 0  Yes = 1 |
| 21.11 | Anemia | No = 0  Yes = 1 |
| 21.12 | Hypoxic Ischemic Encephalopathy | No = 0  Yes = 1 |
| 21.13 | Conjunctivitis | No = 0  Yes = 1 |
| 21.14 | Acute kidney injury | No = 0  Yes = 1 |
| 21.15 | Neonatal seizure | No = 0  Yes = 1 |
| 21.16 | subgaleal hemorrhage | No = 0  Yes = 1 |
| 21.17 | MMC | No = 0  Yes = 1 |
| 21.18 | Others | No = 0  Yes = 1 |
|  | If others, specify |  |
|  | If hospital acquired infection, | Blood culture positive  Blood culture negative |
| 22 | Management modalities |  |
| 22.1 | Radiant warmer | No = 0  Yes = 1 |
| 22.2 | Incubator | No = 0  Yes = 1 |
| 22.3 | IV antibiotics | No = 0  Yes = 1 |
| 22.4 | Intranasal oxygen | No = 0  Yes = 1 |
| 22.5 | CPAP | No = 0  Yes = 1 |
| 22.6 | Phototherapy | No = 0  Yes = 1 |
| 22.7 | Exchange transfusion | No = 0  Yes = 1 |
| 22.8 | Aminophylline | No = 0  Yes = 1 |
| 22.9 | Major surgery | No = 0  Yes = 1 |
| 22.10 | Diuretics | No = 0  Yes = 1 |
| 22.11 | Antiepileptics | No = 0  Yes = 1 |
| 22.12 | No management | No = 0  Yes = 1 |
| 22.13 | Suction | No = 0  Yes = 1 |
| 22.14 | Others | No = 0  Yes = 1 |
|  | If others, specify |  |
| 23 | If IV antibiotics, |  |
| 23.1 | Ampicillin | No = 0  Yes = 1 |
| 23.2 | Gentamicin | No = 0  Yes = 1 |
| 23.3 | Cefotaxime | No = 0  Yes = 1 |
| 23.4 | Ciprofloxacin | No = 0  Yes = 1 |
| 23.5 | Vancomycin | No = 0  Yes = 1 |
| 23.6 | Ceftazidime | No = 0  Yes = 1 |
| 23.7 | Meropenum | No = 0  Yes = 1 |
| 23.8 | Metronidazole | No = 0  Yes = 1 |
| 23.9 | Cloxacillin | No = 0  Yes = 1 |
| 23.10 | Ceftriaxone | No = 0  Yes = 1 |
| 23.11 | Azithromycin | No = 0  Yes = 1 |
| 23.12 | Others | No = 0  Yes = 1 |
|  | If others, specify |  |
| 24 | Maintenance fluid | No = 0  Yes = 1 |
|  | If yes, continued for | 24 hrs  24-72 hrs  >72 hrs |
| 25 | Status of feeding | NPO  PO feeding  Unspecified |
| 26 | If on PO feeding, type of feeding | Breast milk  Formula milk  Unspecified |
| 27 | Feeding modality | Direct breast feeding  NGT feeding  Cup feeding  Bottle feeding  Not specified |
| 27.1 | Direct breast feeding | No = 0  Yes = 1 |
| 27.2 | NGT feeding | No = 0  Yes = 1 |
| 27.3 | Cup feeding | No = 0  Yes = 1 |
| 27.4 | Bottle feeding | No = 0  Yes = 1 |
| 27.5 | Not specified | No = 0  Yes = 1 |
| 28 | Amount of feeding | Full feeding  Trophic feeding |
| 29 | Survival | Censored  Died |
| 30 | Outcome | Censored = 0  Died = 1 |
| 31 | Follow up time in days |  |
| 32 | If died, immediate cause of death |  |
| 33 | If died, age at time of death in days |  |
| 34 | If dies, duration of stay at the NICU of ZMH before death in days |  |
| 35 | Outcome if censored | Discharged  Left against medical advice  Disappeared from ward  Referred |
|  | If discharged, age at discharge in days |  |
|  | If discharged, total duration of hospital stay in days |  |
|  | If left against medical advice, age at the time of event in days |  |
|  | If left against medical advice, total duration of hospital stay in days |  |
|  | If disappeared from ward, age at time of event in days |  |
|  | If disappeared from ward, total duration of hospital stay in days |  |
|  | If referred out, age at time of event in days |  |
|  | If referred out, total duration of hospital stay in days |  |
