# Supplementary material for: Survival and predictors of neonatal mortality: a hospital-based retrospective follow-up study from Addis Ababa, Ethiopia
Source: PeerJ. 2026 Jul 22;14:e21473. doi: 10.7717/peerj.21473 (PMC13401358; doi:10.7717/peerj.21473)
Supplement: Supplemental Information 3 [file peerj-14-21473-s003.docx]

STROBE Statement—checklist of items that should be included in reports of observational studies

|  | **Item**  **No** | **Recommendation** |
| --- | --- | --- |
| **Title and abstract** | 1 | 1. Indicate the study’s design with a commonly used term in the title or the abstract   **Title: "A retrospective cohort study..."** |
|  |  | (*b*) Provide in the abstract an informative and balanced summary of what was done  and what was found  **Abstract: Clearly outlines objectives, methods, results, and conclusions. Title includes "retrospective cohort study"; abstract is structured** |
| **Introduction** |  |  |
| Background/rationale | 2 | Explain the scientific background and rationale for the investigation being reported  **The study provides a clear scientific background and rationale** |
| Objectives | 3 | State specific objectives, including any prespecified hypotheses  **Objectives explicitly stated in the introduction.** \| |
| **Methods** |  |  |
| Study design | 4 | Present key elements of study design early in the paper  **Design explicitly stated in the first line of Methods section of Abstract.** |
| Setting | 5 | Describe the setting, locations, and relevant dates, including periods of recruitment,  exposure, follow-up, and data collection  **Methods: Study period (Sept 2020–Sept 2023), location (Zewditu Memorial Hospital), eligibility (all neonates admitted to NICU). Dates, setting, and inclusion criteria clearly defined.** |
| Participants | 6 | 1. *Cohort study*—Give the eligibility criteria, and the sources and methods of selection of participants. Describe methods of follow-up   **Eligibility Criteria:**  **Inclusion: Neonates admitted to the Neonatal Intensive Care Unit (NICU) within 28 days of birth.**  **Exclusion: Neonates with lost charts**  **Sources and Selection:**  **Participants were selected from all admissions to the NICU of Zewditu Memorial Hospital between Sept 11th, 2020- Sept 10th, 2023 by using systematic random sampling. Data were extracted from patient charts and admission logs.**  **Follow-up Methods:**  **Neonates were followed from admission until discharge, transfer, death, or 28 days post-birth (whichever occurred first).** |
| Variables | 7 | Clearly define all outcomes, exposures, predictors, potential confounders, and effect  modifiers. Give diagnostic criteria, if applicable  **Methods: Predictors (birth weight, sepsis, RDS, etc.), outcome (neonatal mortality), confounders (adjusted in Cox model). All variables explicitly defined in Tables and Methods.** |
| Data sources/ measurement | 8* | For each variable of interest, give sources of data and details of methods of assessment (measurement).  **Source of data is clearly stated to be patient medical records and all variables were taken as they were documented in the patients’ medical records** |
| Bias | 9 | Describe any efforts to address potential sources of bias  **Multivariate analysis was done to reduce confounding bias**  **Missingness of charts was assumed to be random, to reduce selection bias** |
| Study size | 10 | Explain how the study size was arrived at  **Described in the method section**  **For predictors of neonatal mortality risk, the sample size was calculated using Cox method, with variability of 0.5, 95% confidence interval, 5% margin of error, 5% contingency. Failure probability of 0.0192 was taken from a previous study done in Tikur Anbessa Hospital. The final sample size that was selected for this study was 1,150.** |
| Quantitative variables | 11 | Explain how quantitative variables were handled in the analyses. If applicable,  describe which groupings were chosen and why  **Quantitative variables were categorized based on clinical and epidemiological relevance:**  **Birth weight: Grouped as <1,000 g (ELBW), 1,000–1,499 g (VLBW), 1,500–2,499 g (LBW), and ≥2,500 g (NBW) per WHO standards to align with mortality risk strata.**  **Age at admission: Analyzed continuously (median/IQR) and categorically (≤24 hrs vs. >24 hrs) to capture early critical periods.**  **Temperature: Dichotomized (<36.5°C vs. ≥36.5°C) to assess hypothermia’s impact.**  **Follow-up time: Treated continuously (days) in survival analyses, censored at discharge/28 days.** |
| Statistical methods | 12 | (*a*) Describe all statistical methods, including those used to control for confounding **Methods: Cox regression, Kaplan-Meier, log-rank tests; missing data handled via exclusion. Statistical methods thoroughly described.** |
|  |  | 1. Explain how missing data were addressed   **Missing data were handled as follows:**  **Missing patient charts: Entirely excluded from the study**  **Missing variables within included patients: Complete case analysis as missingness was minimal (<5% for all key variables) and assumed to be random.** |
|  |  | (*d*) *Cohort study*—If applicable, explain how loss to follow-up was addressed  **Not applicable as this study is a retrospective cohort study** |
|  |  | (*e*) Describe any sensitivity analyses |
|  |  | **No sensitivity analyses were done** |

| **Results** |  | |
| --- | --- | --- |
| Participants | 13* | 1. Report numbers of individuals at each stage of study—eg numbers potentially eligible, examined for eligibility, confirmed eligible, included in the study, completing follow-up, and analysed   **Numbers of eligible and number of missing charts stated** |
|  |  | 1. Give reasons for non-participation at each stage   **Not Applicable** |
|  |  | 1. Consider use of a flow diagram   **Flow diagram used Figure 1 to describe the systematic random sampling technique** |
| Descriptive  data | 14* | (a) Give characteristics of study participants (eg demographic, clinical, social) and information  on exposures and potential confounders  **The study reports demographic and clinical characteristics of neonates (e.g., birth weight categories, diagnoses like sepsis, PNA, RDS), exposures (e.g., low birth weight), and confounders (e.g., gestational age, sepsis status).** |
|  |  | 1. Indicate number of participants with missing data for each variable of interest   **For included cases, we employed complete case analysis, excluding records only from analyses involving specific missing variables while retaining them for other analyses where data were complete. Missingness was minimal, <5% for all key variables, and assumed to be random.** |
|  |  | 1. *Cohort study*—Summarise follow-up time (eg, average and total amount)   **Follow up time explicitely described as total follow-up time (7,945 neonate-days)** |
| Outcome data | 15* | *Cohort study*—Report numbers of outcome events or summary measures over time  **Total follow-up time (7,945 neonate-days) and outcome events (109 deaths, 83.49% within 7 days) are reported**. |
| Main results | 16 | (*a*) Give unadjusted estimates and, if applicable, confounder-adjusted estimates and their precision (eg, 95% confidence interval). Make clear which confounders were adjusted for and  why they were included  **Unadjusted estimates (e.g., mortality rates by birth weight) and adjusted estimates (e.g., AHRs for sepsis, PNA) with 95% CIs are provided. Confounders (e.g., birth weight, sepsis) are clearly stated.** |
|  |  | 1. Report category boundaries when continuous variables were categorized   **Birth weight categories (<1,000g, 1,000–1,499g, etc.) are reported with boundaries.** |
|  |  | (*c*) If relevant, consider translating estimates of relative risk into absolute risk for a meaningful  time period  **Absolute risk translation is not provided.** |
| Other analyses | 17 | Report other analyses done—eg analyses of subgroups and interactions, and sensitivity  Analyses  **No subgroup, interaction, or sensitivity analyses were done.** |
| **Discussion** |  |  |
| Key results | 18 | Summarise key results with reference to study objectives  **Key results (e.g., predictors of mortality, early neonatal deaths) are summarized with reference to objectives.** |
| Limitations | 19 | Discuss limitations of the study, taking into account sources of potential bias or imprecision.  Discuss both direction and magnitude of any potential bias  **Limitations include potential bias from missing patient charts, temporal bias due to its retrospective chart review design, and reliance on neonatal medical records as the primary source of data which are inadequate for capturing maternal information were discussed** |
| Interpretation | 20 | Give a cautious overall interpretation of results considering objectives, limitations, multiplicity  of analyses, results from similar studies, and other relevant evidence  **Results are interpreted cautiously, with comparisons to similar studies (e.g., Ethiopian and regional data) and discussion of implications for NICU care.** |
| Generalisability | 21 | Discuss the generalisability (external validity) of the study results  **External validity is addressed (e.g., applicability to tertiary hospitals in similar low-resource settings, with caveats about rural-urban differences).** |

**Other information**

Funding 22 Give the source of funding and the role of the funders for the present study and, if applicable, for the original study on which the present article is based

**Not applicable**
